# Patient-Reported Experience Measurements From Individuals With Inherited Retinal Disorders Involved in Observational Research

**DOI:** 10.1167/tvst.13.12.9

**Published:** 2024-12-06

**Authors:** Malena Daich Varela, Shaima Hashem, Dayyanah Sumodhee, Michel Michaelides

**Affiliations:** 1 Moorfields Eye Hospital, London, UK; 2 UCL Institute of Ophthalmology, University College London, London, UK

**Keywords:** inherited, retinal, disorders, patient, experience

## Abstract

**Purpose:**

Inherited retinal disorders (IRD) are a complex group of conditions. By developing the first patient-reported experience measurement (PREM) questionnaire tailored for individuals with IRD participating in natural history studies, we gathered information on individuals’ views of their experience while they are involved in research.

**Methods:**

Adults with IRD who (i) were enrolled in a natural history study taking place at Moorfields Eye Hospital (London, UK), (ii) had attended at least two study visits, (iii) the most recent one being less than two weeks before the questionnaire, and (iv) who were not involved in interventional research, were considered for participation.

**Results:**

Fifty individuals completed the PREM questionnaire at a mean age of 31.1 ± 11 years old and were diagnosed at a mean age of 14 ± 9.7 years old. Most individuals rated “getting closer to receiving treatment’ as their main motivation to enroll in the study, and their biggest influence was their own curiosity. Individuals were more satisfied with the care they received, and least satisfied with the efficiency of the visit. After validity and reliability assessments, the final PREM was created, with 27 questions and five sections, and Cronbach alpha coefficient between 0.316 and 0.756 in each section.

**Conclusions:**

The PREM instrument allowed us to assess the overall satisfaction of individuals with IRD involved in research, detect possible barriers to research participation, and ways of improving our care.

**Translational Relevance:**

The final version can be included in future research and other sites worldwide, to maintain high quality standards.

## Introduction

Inherited retinal disorders (IRD) are a complex group of conditions with a wide genotypic and phenotypic spectrum.^1^^–^^4^ These rare diseases are characterized by a suboptimal visual experience which can significantly impact individuals’ ability to function and decrease their quality of life.^5^ They can be stationary or progressive, and individuals are generally diagnosed from birth/childhood to early adulthood.^6^ There are broadly four types of IRD: rod-cone dystrophy (also known as retinitis pigmentosa), early onset severe retinal dystrophy (EOSRD), cone-rod dystrophy (CRD), and macular dystrophy (MD). Approximately 1 in 2000 individuals worldwide is affected by this group of disorders,^7^ and they are the commonest cause of blindness in the working-age population in England.^8^

There are ongoing efforts towards improving the care of individuals with rare diseases,^9^^–^^11^ and research focusing on IRD is ever-growing, with one gene supplementation therapy already approved for *RPE65*-EOSRD.^12^ There are numerous ongoing observational studies and clinical trials for individuals with IRD taking place worldwide. Usually, individuals hear about these initiatives through patient associations or are invited to take part in research via their routine clinical care appointments. Individuals who participate in observational “natural history’ studies get to (i) be part of the clinical trial development, (ii) undergo thorough evaluations which enable discussions about disease progression and deeply characterize their retinal phenotype, and (iii) potentially become eligible for future interventional trials. Yet, patient enrolment in an observational study can sometimes be an act of selflessness where they volunteer their time with the aim of contributing to science and helping future generations, rather than a true gain for themselves.

Several standardized questionnaires have been developed to assess particular aspects of individuals’ visual experience. These instruments have shown significant reliability and validity and are included in research settings as patient-reported outcome measures (PROM).^13^ They have recently gained importance due to becoming key outcome measures in gene therapy clinical trials.^14^^–^^16^ Different questionnaires exist to assess adults and children visual experience.^17^ Deepening our understanding of patient perspectives is helpful in developing measurement strategies that can reflect the patient voice in clinical trials.

However, we are lacking an instrument that can tell us how satisfied individuals are with their involvement in research, and how we can improve the quality of care we provide to patients enrolled in observational studies. Patient-reported experience measurements (PREM) are standardized questionnaires developed to gather information on individuals’ views of their experience whilst receiving certain type of care.^18^ These have been developed for a few circumstances so far (e.g. surgical excision of lower limb osteomyelitis, paediatric cataracts), and are indicators of patient care quality.^19^^–^^21^

In this paper, we present the first PREM questionnaire tailored for individuals with IRD who are participating in natural history studies. We also analyse the responses of individuals from Moorfields Eye Hospital (MEH, London, UK), and discuss how this instrument can improve the care we provide and decrease barriers to research participation.

## Methods

This project was approved by the UK Health Research Authority and the local ethics committee (IRAS 314636). To develop the PREM questionnaire, the authors conducted a targeted conceptual literature review, had expert advice meetings to identify relevant concepts for measurement, and implemented a pilot test with five individuals who gave feedback. Eighteen questions (50%) were extracted and adapted from previously validated PREM questionnaires,^21^^–^^23^ while the remaining 18 (50%) were developed specifically for this project and individuals. Informed consent was obtained from all patients and the study honored the tenets of the Declaration of Helsinki.

Multiple natural history studies usually take place at MEH, either focusing in one particular gene (*RPGR*, *RDH12*, i.e.) or a group of conditions (cone disorders). All adult individuals with IRD who were enrolled in natural history studies sponsored by MeiraGTx or the Foundation Fighting Blindness (FFB), taking place at MEH clinical research facility (CRF) were considered for participation. They had to have attended at least two natural history study visits, the most recent one being less than 2 weeks prior to the questionnaire, and not be involved in interventional research. Research visits comprise at least one full day at the CRF, where participants undertake various tests such as visual field, visual acuity, low-luminance visual acuity, and various retinal imaging.

Eligible individuals were presented with study information and were invited to complete the prototype questionnaire once, on their scheduled follow up research visits. If interested in completing the questionnaire, individuals reviewed the patient information sheet and signed the informed consent form. A short demographics form and the questionnaire about their experience thus far was either provided to the individuals to complete on their own or, if preferred, read to them by the researchers (MDV, SH). Individuals were asked to rate their agreement with different statements on a Likert scale “fully agree,” “somewhat agree,” “not sure,” “somewhat disagree,” and “fully disagree.”

Once the target number of individuals was reached (n = 50), the validity and reliability of the prototype PREM questionnaire were assessed with SPSS (software IBM SPSS, version 29; Chicago, IL, USA). There was no missing data (questions not answered by individuals), indicating that all questions were clear to individuals. Questions with an Item Reliability Index (IRI) less than 0.3 and a Cronbach's alpha coefficient less than 0.7 were considered as poor. Inter-item correlation matrix was used to assess the correlation between questions. Questions with suboptimal results or assessing similar aspects of the experience were removed. Validity was also qualitatively assessed by verbally asking individuals whether the questions made sense and changes were made following their feedback. Basic statistics assessing the questionnaire results were done with GraphPad Prism 8.0.2 (GraphPad Software, San Diego, CA, USA). The threshold of significance was set at *P* < 0.05.

## Results

### Demographics

Fifty individuals completed the PREM questionnaire at a mean age of 31.1 ± 11 years old (median 29.5). Forty-two (84%) were male; six (12%) were Asian, and 44 (88%) were white. Thirty (60%) had *RPGR*-related retinal dystrophy, nine (18%) had *ABCA4*-Stargardt disease, five (10%) had *KCNV2*-related retinal dystrophy, three (6%) had *RDH12*-related retinal dystrophy, two (4%) had Achromatopsia, and one (2%) had Usher syndrome. Individuals were diagnosed at a mean age of 14 ± 9.7 years old (median = 12.5). Thirty-two (44%) were working full time, nine (18%) were students, four (8%) were working part time, four (8%) were unemployed, and one (2%) was retired. Twenty (40%) had a graduate degree, 12 (24%) had completed up to GCSE, seven (14%) had postgraduate degrees, six (12%) up to A levels, and five (10%) had some university education. Thirty-three (66%) lived with friends or family, 11 (22%) lived alone, and six (12%) lived with their spouse. For six (12%) individuals, the travel back and forth from their home to MEH took less than one hour, for 10 (20%) between one and two hours, for 14 (28%) between two and four, and for 20 (40%) more than four hours. Twenty-three (46%) people were invited to the natural history study by the research team, 20 (40%) by their routine care doctor, four (8%) heard about it from a friend or family member, three (6%) actively looked for research opportunities, two (4%) saw it on a website, and two (4%) heard it from patient associations.

### Questionnaire

The PREM questionnaire was divided into five sections: (i) communication and information, (ii) efficiency, (iii) patient care, (iv) motivation, and (v) research visit versus regular clinic visit (Table 1; Fig.).

1. On the communication and information section, more than 80% of patients fully agreed with an appropriate message conveyed during the informed consent, with a respectful tone, as much information as needed, and taking into account the visual impairment. The question with the least satisfaction was about providing additional resources that could help support them with their visual impairment (78% agreed this need was met).
2. Regarding efficiency, the majority of patients (86%–94%) were satisfied with the organization of the visit and the waiting time. There were some concerns regarding the scheduling process (24% were not fully satisfied), and most patients (88%) felt fatigued after their research day.
3. Most participants (84%) were satisfied about the care they received during their research visits. More than 80% would recommend enrolling in the study and felt their participation was a good use of their time.
4. Discussing motivation, 80% or more patients understood the tests they were being asked to do and their results and felt proud about their participation in the study, considering it meaningful. Interestingly, only 76% shared their involvement in research with friends and family.

**Table 1.** Summary of Responses to PREM Questionnaire From Patients With Inherited Retinal Disorders Participating in Natural History Studies

	FA (n)	SA (n)	NS (n)	SD (n)	FD (n)
Communication & information					
1. ICF process	47	3	0	0	0
2. Discussed symptoms	44	5	0	1	0
3. Physician listened	49	0	0	0	0
4. As much information as wished	43	5	2	0	0
5. Available support discussed	34	5	5	4	2
6. Appropriate medical check-up	46	3	1	0	0
7. Interest as a whole person	48	2	0	0	0
8. Visual impairment taken into account	46	2	0	2	0
9. Respectful team	48	1	0	0	0
10. Communication was OK	45	3	2	0	0
11. Adequately trained team	46	4	0	0	0
Efficiency					
12. Organized scheduling	38	8	2	1	1
13. Reminded of my visit with time	43	3	2	0	2
14. Efficient organization	45	3	2	0	0
15. CRF appropriate working hours	47	3	0	0	0
16. Acceptable waiting	46	4	0	0	0
17. Rushed	1	4	1	4	40
18. Fatigued	24	20	0	1	5
Patient care					
19. Good use of time	44	4	2	0	0
20. Useful information	42	6	1	1	0
21. Needs were considered	43	6	1	0	0
22. Satisfied with participation	45	3	2	0	0
23. Received the support needed	44	4	2	0	0
24. Recommend study	42	6	2	0	0
Motivation					
25. Understand tests	40	8	0	2	0
26. Test results explained	40	6	1	2	1
27. Meaningful participation	46	3	1	0	0
28. Feel proud	42	5	3	0	0
29. Feel ashamed	0	0	1	1	48
30. Share involvement	38	6	2	2	2
Motivation^*^ (31)					
Know more about retinal condition	7	14	29	—	—
Help science	17	24	9	—	—
Getting closer to treatment	26	12	12	—	—
Influence^*^ (32)					
Friends and family	10	13	26	—	—
Social responsibility	12	21	16	—	—
Personal curiosity	28	15	7	—	—
Research vs. clinic					
33. Satisfied with clinic	31	8	5	5	1
34. Fuller information in research	36	6	6	2	0
35. Clinic visits are better use of time	2	1	17	3	27
36. Enjoy clinic visits more	2	2	12	7	27

A, agree; CRF, clinical research facility; D, disagree; F, fully; ICF, informed consent form; NS, not sure; S, somewhat.

* On these questions the patients were asked to rank most, secondary, and least important motivation (31) and influence (32) to enroll in the research study.

**Figure.**
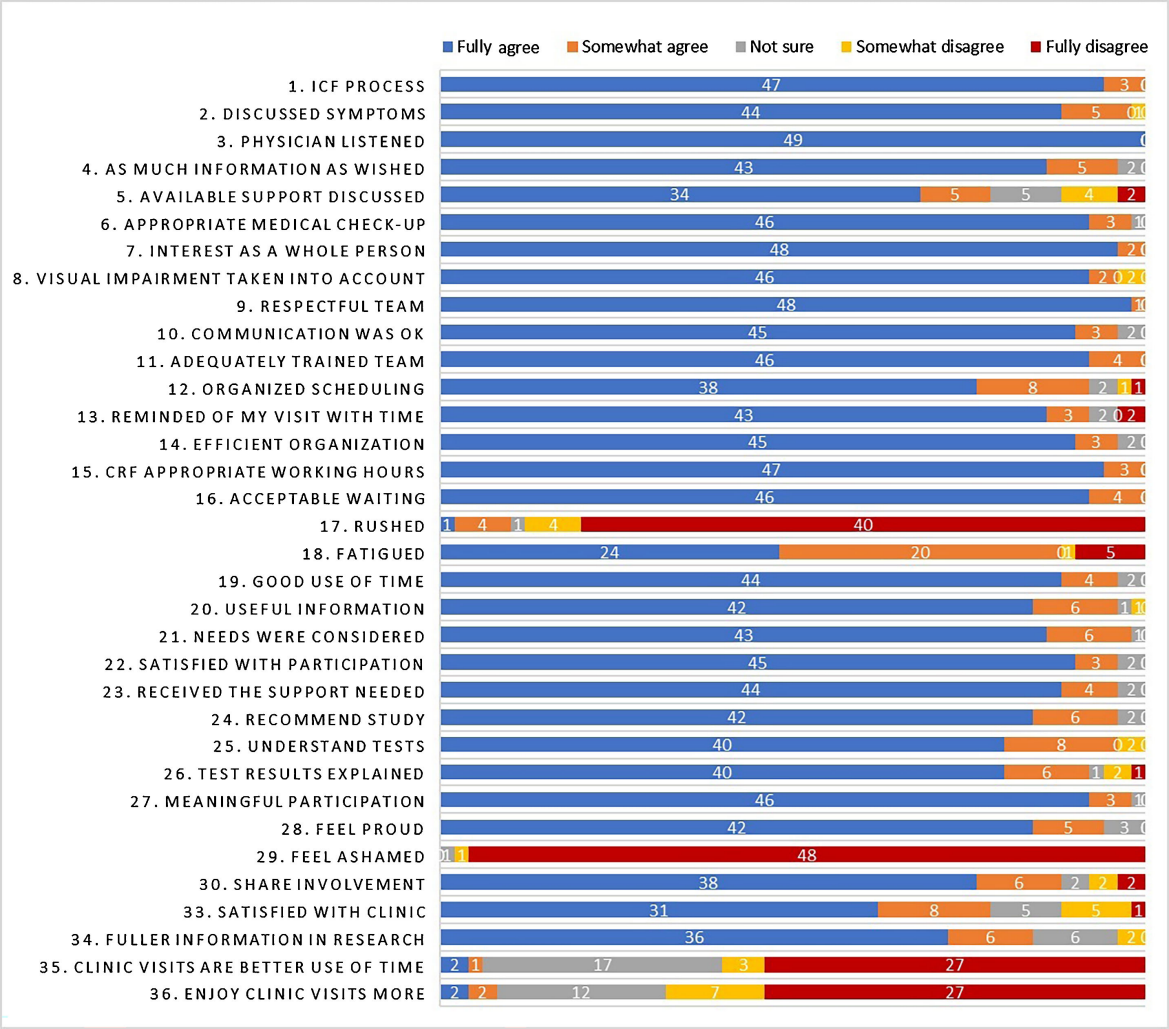
Schematic representation of the PREM questionnaire and answers from the participants. ICF, informed consent form.

Question 31 asked what the patients’ main motivations for enrollment in the research study were. Twenty-six individuals (52%) rated “getting closer to receiving treatment” as their main motivation, 17 (34%) chose “helping science and future generations,” and seven (14%) chose “knowing more about the retinal condition.” The option that was most commonly a secondary motivation was “helping science and future generations,” with 24 (48%) individuals choosing it like this, and “knowing more about the retinal condition” was the weakest motivation for 29 individuals (58%).

Question 32 queried the patients about what the biggest influence on the decision was to take part in research. Twenty-eight (56%) stated it was personal curiosity, for 12 (24%) it was social norm/responsibility, and for 10 (20%) it was conversations with friends and family. The most common second biggest influence was social norm/responsibility for 21 individuals (42%), and the most commonly weakest influence was conversations with friends and family for 26 (52%).

5. The last section compared the research visits with the routine care visits in the clinic. Although most patients (62%) were satisfied with their clinic visits, 72% stated receiving fuller information during the research visits, and 54% considered research visits were a better use of their time and more enjoyable than clinic visits.

Individuals were also invited to comment on anything else if they wished to do so on a free-form textbox, and these opinions have been organized in Table 2. Individuals who felt tired after the research day were asked if they preferred to split the visit in two, and they mostly preferred getting it done in one day.

**Table 2.** Comments at the End of the Questionnaire, on a Free-Form Textbox


Areas of opportunity
“Some tests are difficult to understand”
“It is rather disappointing how long it takes to come up with a treatment”
“You should do more publicity about available research opportunities”
“It is sometimes tough to book clinic appointments”
“Seeing different people on every visit makes it hard to build a trusting relationship”
“Not enough counselling available”
“Lack of empathy in clinic, lack of knowledge by the doctors, and lack of overall support”
“There are sometimes mixed messages from different team members”
“I get lost in building when coming”
“It would be helpful to know more about what has happened since the last visit at the beginning of the research appointment”
“How is my data being analyzed”
Positive outcomes
“I am thankful for being able to participate”
“Good research team”
“The questionnaire was appropriate”

### Associations

Individuals were overall more satisfied with the care they received (Section III of the PREM, with only one answer expressing somewhat disagreement with statements), and least satisfied with the efficiency of the visit (Section II, particularly about feeling tired after the long research day and the scheduling process, 10 answers expressing full and somewhat disagreement with statements).

There was no significant association between age taking the test (*P* = 0.97) or with age at diagnosis (*P* = 0.96) and satisfaction. Although there were not significant differences between any group (*P* = 0.2–0.9), the following patterns were seen. Men had more satisfaction than women; individuals with highest satisfaction lived one to two hours away from Moorfields, and those with least were two to four hours away; individuals with Stargardt disease were the least satisfied, whereas those with *RDH12*-retinal dystrophy were the most satisfied, closely followed by individuals with *RPGR*-retinal dystrophy; individuals with A-level education had the highest satisfaction, whereas those with a postgraduate degree had the least; those working full-time were the most satisfied, whereas those working part-time were the least.

Participants who enrolled in the study to learn more about their retinal condition were, on average, younger (mean age 27 years old) compared to those whose primary motivation was seeking treatment (mean age 31 years old) and those who joined the study to support scientific research (mean age 32.8 years old). Similarly, participants mainly influenced by conversations with friends and family to get involved in research were the youngest (mean age 23.5 years old), whereas those motivated by personal curiosity (mean 31.4 years old) and social responsibility (mean 36.6 years old) were older.

### PREM Assessment

The validation analysis showed that there was no violation of the validation rules in any section. Nine questions (nos. 2, 9, 17, 25, 28, 29, 31, 32, and 34) were deleted after the validity and reliability assessments, leaving a final number of 27 questions and five sections (Supplementary Material). Questions 31 and 32 were removed from the final PREM instrument because we considered them our research interest and not necessarily relevant for individuals’ experiences.

The Cronbach alpha was 0.677 in the communication and information section, 0.555 in the efficiency section, 0.756 in patient care, 0.316 in motivation, and 0.451 in research versus clinic sections. IRI was above 0.3 for all questions in all sections, except for two questions in the motivation section ('Whether test results are explained” (0.039) and “To help Science” (0.027)). These last two questions were deemed as meaningful by both individuals and the research team; hence, they remained in the final questionnaire.

## Discussion

Natural history studies provide invaluable data on diseases, helping delineate prognostic factors and performance thresholds, which aid the development of outcome measures and appropriate endpoints, improving patient selection and leading to more efficient clinical trials and patient care.^24^ There are currently more than 200 natural history studies in ophthalmology worldwide, with nearly half of them focused on retinal diseases, and a quarter on IRD (229 total, 98 for retina, 52 for IRD; www.clinicaltrials.gov, queried on February 2024). The development of the first PREM questionnaire adapted for individuals with IRD, with good validity and reliability, has enabled us to analyze the experience of participating individuals at the state-of-the-art clinical research facility of MEH, shedding light on possible barriers and areas of opportunity to research participation for individuals living with IRD.

A rather expected finding of this work was that the main motivation of individuals to enroll in observational studies was to get closer to receiving treatment; however, this was not the most common option among the youngest individuals (whose main drive was to know more about their condition). Having in mind the possible different motivations, the invitations to take part could be tailored to different populations, increasing the chances of engaging their participation. It is possible that the younger patients can cope with the visual impairment and do not feel the need of engaging in experimental treatments, whereas older patients (likely with larger career and family responsibilities) are more compelled toward an intervention that may halt disease progression. While inviting individuals into research, it is important to highlight the pace at which treatments may get developed to manage their expectations.^25^

Although most individuals agreed they preferred a longer one-day visit instead of two half days, an allocated room to rest and recharge could be a valuable addition to the comfort, also improving access to support services. The addition of “wellbeing suites” closer to the research facility, where individuals could connect, talk to support services, nap, or do workshops such as yoga or meditation, has been found to promote psychologically supportive environments.^26^ Another interesting finding of this project was how some individuals reported that the common rotation of doctors and clinical trial coordinators affected their overall experience, having suboptimal communication with the research team. This highlighted the importance of trying to ensure smooth transitions when research staff change to optimize the individuals experience in return for their valuable time and commitment to observational studies.

Last, in current times where remote monitoring is becoming increasingly accessible and standardized,^27^^–^^29^ it may be possible to revisit the concept of traditional natural history studies to include real-world data, potentially lowering their cost and arguably providing more meaningful data.^30^ For individuals coming both to clinic and research visits, we may consider using standard-of-care data and providing them with more comprehensive clinic visits instead of two separate visits, where there is overlap with assessments and protocols of acquisition. There is a need to lower the costs of developing complex therapies, and using this real-world data may help.

In conclusion, the PREM instrument allowed us to assess the overall satisfaction of individuals with IRD involved in research, and detect possible barriers to research participation (e.g., tiredness, and frequent change of staff), and ways of improving our care (e.g., more discussion about available support, providing more information about research status). The final version can be included in future research and other sites worldwide to monitor the individuals’ experiences and maintain high-quality standards.

## Supplementary Material

Supplement 1
