# Supplementary material for: Patient-Reported Experience Measurements From Individuals With Inherited Retinal Disorders Involved in Observational Research
Source: Transl Vis Sci Technol. 2024 Dec 6;13(12):9. doi: 10.1167/tvst.13.12.9 (PMC11629914; doi:10.1167/tvst.13.12.9)
Supplement: Supplement 1 [file tvst-13-12-9_s001.docx]

Patient-reported experience measurements from individuals with Inherited Retinal Disorders involved in observational research Questionnaire

Communication and information

|  | **1** (fully agree) | **2** (somewhat agree) | **3**  (not sure) | **4** (somewhat disagree) | **5**  (fully disagree) |
| --- | --- | --- | --- | --- | --- |
| I am satisfied with the informed consent process (I was given time to read through the consent form, ask questions and had them answered satisfactorily). |  |  |  |  |  |
| The physician listened to me and answered my questions in a timely and clear manner. |  |  |  |  |  |
| I got as much information as I wished about my condition. |  |  |  |  |  |
| I was also given information about available opportunities, counselling and/or support for me. |  |  |  |  |  |
| The physician did appropriate inquiring and medical check-up (discussion and evaluation) to better understand my condition. |  |  |  |  |  |
| The physician showed interest in me as a whole person and understood my concerns. |  |  |  |  |  |
| My visual impairment was taken into account during my visit. |  |  |  |  |  |
| The communication between me and the healthcare team was satisfactory. |  |  |  |  |  |
| The healthcare team was adequately trained to do the assessments they were in charge of during my visit. |  |  |  |  |  |

Efficiency

|  | **1** (fully agree) | **2** (somewhat agree) | **3**  (not sure) | **4** (somewhat disagree) | **5**  (fully disagree) |
| --- | --- | --- | --- | --- | --- |
| The scheduling process was organized, with options offered to me, and alternatives contemplated. |  |  |  |  |  |
| I was reminded of my visit with enough time to organize my work and family commitments. |  |  |  |  |  |
| I feel the organization between the different care units (study coordinators, optometry, nurses, physicians) was efficient. |  |  |  |  |  |
| I think the available working hours of the Clinical Research Facility are appropriate. |  |  |  |  |  |
| The time in the waiting room was acceptable. |  |  |  |  |  |
| I felt fatigued after my visit. |  |  |  |  |  |

Patient care

|  | **1** (fully agree) | **2** (somewhat agree) | **3**  (not sure) | **4** (somewhat disagree) | **5**  (fully disagree) |
| --- | --- | --- | --- | --- | --- |
| This was a good use of my time. |  |  |  |  |  |
| I was provided useful information regarding my condition. |  |  |  |  |  |
| My needs and expectations were considered. |  |  |  |  |  |
| I am satisfied with my participation in this natural history study. |  |  |  |  |  |
| I received the overall support that I expected/need. |  |  |  |  |  |
| I would recommend enrolling into this study. |  |  |  |  |  |

Motivation

|  | **1**  (fully agree) | **2** (somewhat agree) | **3**  (not sure) | **4** (somewhat disagree) | **5**  (fully disagree) |
| --- | --- | --- | --- | --- | --- |
| The test results and any change over time was explained. |  |  |  |  |  |
| I think participating in the natural history study is meaningful. |  |  |  |  |  |
| I share my involvement in research with my friends and family. |  |  |  |  |  |

**Research visit versus regular clinic visit**

|  | **1** (fully agree) | **2** (somewhat agree) | **3** (not sure) | **4** (somewhat disagree) | **5** (fully disagree) |
| --- | --- | --- | --- | --- | --- |
| I am satisfied with the information and care I receive during my **regular clinic visits**. |  |  |  |  |  |
| Clinic visits are a better use of my time than research visits. |  |  |  |  |  |
| I enjoy my clinic visits more than the research visits. |  |  |  |  |  |

**Any other comment(s) or suggestion(s)?**
